# Multi-Model Machine Learning for Survival Predictions for Castration-Resistant Prostate Cancer

**DOI:** 10.3390/cancers18121866

**Published:** 2026-06-07

**Authors:** Tae Jin Kim, Jaeyun Jeong, Young Jin Ahn, Kwang Suk Lee, Jong Soo Lee, Seung Hwan Lee, Won Sik Ham, Byung Ha Chung, Jeong Hyun Lee, Kyo Chul Koo

**Affiliations:** 1 Department of Urology, CHA University Ilsan Medical Center, CHA University School of Medicine, Goyang 10414, Republic of Korea; tjkim81@cha.ac.kr; 2 Bithumb, Seoul 06234, Republic of Korea; jayhey@bithumbcorp.com; 3 Department of Urology, Gangnam Severance Hospital, Yonsei University College of Medicine, Seoul 06273, Republic of Korea; curio2@yuhs.ac (Y.J.A.); calmenow@yuhs.ac (K.S.L.); chung646@yuhs.ac (B.H.C.); 4 Department of Urology, Severance Hospital, Yonsei University College of Medicine, Seoul 03722, Republic of Korea; js1129@yuhs.ac (J.S.L.); leeseh@yuhs.ac (S.H.L.); uroham@yuhs.ac (W.S.H.)

**Keywords:** machine learning, prediction algorithms, prostatic neoplams, castration-resistant, survival

## Abstract

**Background:** Accurate survival prediction is essential for optimizing treatment planning in patients with castration-resistant prostate cancer (CRPC). However, traditional statistical models often underperform because of limited variable inclusion and an inability to account for complex, multidimensional data interactions. **Methods:** We retrospectively collected 46 clinical, laboratory, and pathological variables from 801 patients with CRPC, covering the disease course from initial diagnosis to CRPC progression. Multiple machine learning (ML) models, including random survival forests (RSF), XGBoost, LightGBM, and logistic regression, were developed to predict cancer-specific mortality (CSM), overall mortality (OM), and 2- and 3-year survival status. The dataset was divided into training and test cohorts (80:20), and 10-fold cross-validation was performed. Performance was assessed using the C-index for regression models and the area under the curve (AUC), accuracy, precision, recall, and F1-score for classification models. Model interpretability was evaluated using SHapley Additive exPlanations (SHAP). **Results:** Over a median follow-up of 24 months, 70.6% of patients experienced CSM. Although XGBoost with its own imputation method achieved the highest C-index in the validation set, RSF demonstrated more stable performance and achieved the highest C-index in the held-out test set for both CSM (0.772) and OM (0.771). For classification tasks, RSF demonstrated superior performance in predicting 2-year survival, whereas XGBoost achieved the highest F1-score for 3-year survival prediction. SHAP analysis identified time to first-line CRPC treatment, hemoglobin level, and alkaline phosphatase level as key predictors of survival outcomes. **Conclusions:** RSF demonstrated robust test-set performance for time-to-event prediction, whereas XGBoost showed complementary value for 3-year survival classification. These models provide accurate and interpretable prognostic tools that may support personalized treatment strategies. External validation and integration of emerging therapies are warranted to enhance broader clinical applicability.

## Simple Summary

Castration-resistant prostate cancer is an advanced stage of prostate cancer with highly variable outcomes, making accurate prognosis important for treatment planning. Many existing prediction algorithms rely on a limited number of variables and may not fully reflect real-world clinical complexity. We analyzed data from 801 patients and developed machine learning models to predict mortality risk and 2- and 3-year survival using clinical, laboratory, and pathological variables collected throughout the disease course. Key predictors included time to first-line treatment after the development of castration resistance, hemoglobin level, and alkaline phosphatase level. Overall, the machine learning models demonstrated better predictive performance than conventional methods. These results may help clinicians provide more individualized prognostic estimates and support treatment discussions with patients.

## 1. Introduction

Advanced prostate cancer (PCa) can initially be managed effectively with androgen deprivation therapy (ADT); however, the majority of patients eventually develop castration resistance [1]. The introduction of multiple lines of systemic therapies, including novel androgen receptor pathway inhibitors, taxane-based chemotherapies, radiopharmaceuticals, and poly (ADP-ribose) polymerase inhibitors, has improved the overall survival of patients with castration-resistant PCa (CRPC) [2,3,4]. Nonetheless, the prognosis remains poor and heterogeneous across different patient subgroups.

With the expansion of treatment options for CRPC, there is a growing need for accurate survival prediction to optimize treatment sequencing. However, traditional statistical methods fail to account for the nonlinear and multidimensional associations among CRPC biological factors and the multiple sequential therapies used throughout the disease course. Several studies have explored prognostic models for predicting overall survival (OS) in patients with CRPC [5,6,7,8]. However, the reported predictive performances were modest, ranging from 0.62 to 0.79, presumably because of limited sample sizes, restricted clinicopathological variables included in the analyses, and reliance on traditional statistical methods that assessed outcomes based on arbitrarily defined risk groups. Indeed, the performance of survival prediction models could potentially be improved by including larger patient cohorts, incorporating a broader range of input variables, and applying machine learning (ML) techniques [5,9]. Recent studies have further highlighted the growing role of ML-based predictive modeling and interpretable artificial intelligence approaches in oncologic prognostication and clinical decision-making across various malignancies.

ML has recently gained popularity in survival prediction because of its ability to process complex, high-dimensional datasets, thereby enabling highly accurate predictive modeling [10,11,12]. Compared with traditional statistical approaches, such as Cox proportional hazards models or Kaplan–Meier analysis, ML methods have demonstrated superior predictive accuracy, particularly in scenarios involving numerous variables [13]. Studies across various cancer types have reported that ML models can achieve predictive accuracies ranging from approximately 70% to 90% and root mean square errors below 20 in regression-based survival analyses [14,15]. Despite these promising outcomes, ML techniques have not yet been extensively applied to datasets specific to patients with CRPC. Given the heterogeneity and therapeutic complexity of CRPC, the application of ML may provide more individualized and precise survival estimates.

This study aimed to develop and compare multiple ML models to predict time to cancer-specific mortality (CSM), overall mortality (OM), and 2- and 3-year survival status after CRPC diagnosis using a comprehensive set of demographic and clinicopathological variables. We aimed to identify the most accurate and reliable ML model to guide clinical decision-making.

## 2. Materials and Methods

### 2.1. Data Collection

Clinical, laboratory, and pathological data comprising 46 variables at the time of initial PCa diagnosis and at the time of progression to CRPC were retrospectively collected from 801 consecutive patients diagnosed with CRPC at two institutions from January 2005 to February 2022. CRPC was defined according to the Prostate Cancer Working Group 2 criteria. Patients were excluded if clinical data were incomplete, if treatment deviated from standard recommendations, or if the cause of death or survival status could not be identified.

Data on CRPC treatments were collected, including the type of therapeutic agent (abiraterone acetate, enzalutamide, cabazitaxel, docetaxel, and olaparib) and the durations of first, second, and third lines of treatment until disease progression. The sequence of administered agents was determined by physician discretion and patient preference. Treatment regimens included intravenous docetaxel (75 mg/m^2^) and cabazitaxel (20 mg/m^2^) administered every three weeks in combination with oral prednisone (5–10 mg), enzalutamide (160 mg), abiraterone (1000 mg) combined with prednisolone (5–10 mg), and olaparib (300–600 mg). Each line of treatment was maintained until disease progression, the development of unacceptable toxicity, or patient refusal.

Survival status and cause of death were determined using data from the National Cancer Registry Database or institutional medical records. Deaths were attributed to CRPC if there was documented progression of metastatic CRPC or if death resulted from treatment-related complications.

### 2.2. Study Endpoints

The primary endpoint of this study was to develop ML models predicting time-to-CSM, time-to-OM, and 2-year and 3-year survival status following the diagnosis of CRPC. The secondary endpoint was to evaluate the discriminative performance of the developed models.

### 2.3. Statistical Analyses

#### 2.3.1. Data Processing

To prepare the dataset, several key variables were derived, including the time interval (months) between CRPC diagnosis and either death or last follow-up, %PSA changes from initial PCa diagnosis to ADT initiation, durations and %PSA changes between PCa diagnosis and ADT initiation, risk-group stratification based on LATITUDE (high-risk) and CHAARTED (high-volume) criteria, and neutrophil-to-lymphocyte ratio. For survival outcome classification, 2-year and 3-year survival status after CRPC diagnosis were encoded as binary variables.

Cases with missing outcome data were excluded before model development. After feature derivation, the final dataset was randomly divided into training and held-out test sets using an 80:20 split prior to any preprocessing procedure to prevent data leakage. A detailed preprocessing and model development workflow is provided in Supplementary Figure S1.

All preprocessing procedures, including imputation, categorical encoding, and scaling (when required), were fitted exclusively on the training set and subsequently applied to the validation/test data using transform-only procedures. Hyperparameter optimization and cross-validation were also conducted entirely within the training set.

Missing data were addressed using imputation methods based on variable type. Continuous variables were imputed using IterativeImputer with a BayesianRidge estimator (scikit-learn version 1.8.0) (max_iter = 20, initial_strategy = “mean”, sample_posterior = False, random_state = 42). Categorical variables were imputed using the most frequent category via SimpleImputer (strategy = “most_frequent”). Categorical variables were subsequently encoded using OneHotEncoder (handle_unknown = “ignore”).

A supplementary missingness table summarizing variable type, number and percentage of missing values, imputation method, and model inclusion status for all variables was additionally provided (Supplementary Table S4). Among the 78 total variables, 60 contained missing values, with a median missingness rate of 1.5%. Fifty variables were ultimately included in the final predictive models.

Some later-line treatment variables demonstrated structural missingness because many patients did not receive those treatment lines. Furthermore, post-CRPC treatment variables and outcome-related variables were excluded from baseline prediction modeling to avoid future information leakage. Date variables were not directly imputed and were used only for interval derivation when necessary.

#### 2.3.2. Model Development

Time-to-event outcomes and binary survival status were modeled using survival (time-to-event) modeling and classification approaches, respectively. For the survival models, the outcome was defined as the time in months from CRPC diagnosis to CSM or OM, with censoring at the last follow-up. For the classification models, binary outcomes represented 2-year and 3-year survival status after CRPC diagnosis.

Survival models were developed using Cox proportional hazards modeling, random survival forests (RSF), and XGBoost-based survival modeling. Classification models were developed using logistic regression, Light Gradient Boosting Machine (LightGBM), XGBoost, and random forest algorithms. Model hyperparameters were optimized within the training set using 10-fold cross-validation. Supplementary Tables S1 and S2 provide details of the applied survival and classification models, including corresponding hyperparameters and search ranges. Hyperparameter optimization was implemented using Optuna in Python (version 3.13.9). The best-performing model in each task category was selected as the final predictive model.

For XGBoost-based survival analysis, the native xgboost.train API with the survival:cox objective was used rather than the accelerated failure time (AFT) objective. Censoring information was incorporated according to the XGBoost Cox convention, in which observed events were encoded as positive survival times and right-censored observations as negative survival times. Hyperparameter optimization was performed using Optuna with a Tree-structured Parzen Estimator (TPE) sampler within the training set. Early stopping was applied using an internal validation split with early_stopping_rounds = 50, and the maximum number of boosting rounds was set to 1000.

Two XGBoost survival variants were evaluated: (1) XGBoost using the predefined external preprocessing/imputation pipeline, and (2) XGBoost with its own internal missing-value handling. Detailed implementation settings, final hyperparameters, and proportional hazards assumption assessment results are summarized in Supplementary Table S5.

For the Cox proportional hazards model, proportional hazards (PH) assumptions were additionally assessed using rank-transformed Schoenfeld residual tests implemented through lifelines.CoxPHFitter.

All analyses were conducted in Python using fixed random seed settings (random_state = 42) to improve reproducibility. Hyperparameter optimization was performed using Optuna within the training set. Major libraries included scikit-learn, xgboost, lifelines, scikit-survival, and shap. Detailed preprocessing workflows, implementation settings, and final hyperparameters are summarized in the Supplementary Materials.

#### 2.3.3. Model Performance Interpretation

Regression models, which generated survival time predictions, were evaluated using Harrell’s concordance index (C-index) to assess discriminative performance. For the classification models, which produced categorical survival status predictions, performance was evaluated using accuracy, area under the receiver operating characteristic curve (AUC), mean precision, mean recall, and F1-score.

To enhance interpretability, the final models were analyzed using the SHapley Additive exPlanations (SHAP) framework. SHAP quantifies the contribution of each input variable to model predictions, enabling understanding of feature importance behind individual predictions.

### 2.4. Ethical Consideration

This study was approved by the Institutional Ethics Committee of Yonsei University Health System (approval number: 3-2016-0190) following a review of the study protocol. All procedures were conducted in accordance with the ethical standards of the Declaration of Helsinki and its most recent revision.

## 3. Results

### 3.1. Patient Characteristics

Baseline demographic and clinicopathological characteristics of the patients at the time of initial PCa diagnosis and progression to CRPC are presented in Table 1. Over a median follow-up period of 24.0 months (interquartile range: 12.0–43.0 months), 566 cancer-specific deaths (70.6%) and 588 overall deaths (73.4%) were observed. The types and distributions of systemic agents administered according to treatment line are provided in Supplementary Table S3.

**Figure 1:**
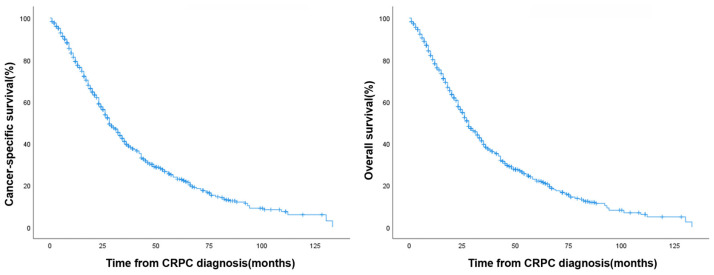
Kaplan–Meier curves for cancer-specific survival and overall survival.

The 2-year and 3-year cancer-specific survival rates were 56.5% and 37.2%, respectively, whereas the 2-year and 3-year overall survival rates were 54.3% and 34.3% (Table 2). These survival outcomes were generally consistent with previously published real-world data on CRPC prognosis [16,17,18,19,20,21]. Kaplan–Meier curves for cancer-specific and overall survivals were generated, with time defined as the interval from CRPC diagnosis to death or last follow-up (Figure 1).

Comparisons between groups were performed using Welch’s *t*-test for continuous variables and the chi-square test for categorical variables. All tests were two-sided, and *p*-values were reported accordingly.

### 3.2. Comparison of Model Performance

Table 3 presents model performance on the test dataset comprising 160 patients. Among the evaluated models, RSF demonstrated strong performance for both CSM and OM prediction. In the validation cohort, XGBoost with internal imputation achieved the highest C-index for both outcomes: 0.771 for CSM (95% CI 0.706–0.836) and 0.773 for OM (95% CI 0.708–0.838). RSF ranked second in the validation set, with C-index values of 0.764 for CSM (95% CI 0.698–0.830) and 0.771 for OM (95% CI 0.706–0.836).

However, in the test set, RSF outperformed all other models, achieving the highest C-index for both CSM (0.772, 95% CI 0.707–0.837) and OM (0.771, 95% CI 0.706–0.836). XGB ranked third, with C-index values of 0.753 (95% CI 0.686–0.820) for CSM and 0.765 (95% CI 0.699–0.831) for OM, respectively. These findings suggested a tendency toward overfitting, as the test-set performance was lower than the validation-set performance. From the perspective of model generalizability, RSF appeared to provide more robust and reliable performance across datasets.

Calibration and clinical utility analyses were additionally performed for the final RSF survival models. Calibration plots for 2- and 3-year OM and CSM predictions demonstrated reasonable agreement between predicted and observed mortality risks in the held-out test set (Supplementary Figure S2). Time-dependent Brier scores ranged from 0.155 to 0.171, whereas integrated Brier scores ranged from 0.162 to 0.166 (Supplementary Table S6), supporting acceptable overall prediction accuracy and calibration performance.

Decision curve analysis further demonstrated favorable net benefit of the RSF models across a broad range of clinically relevant threshold probabilities compared with treat-all and treat-none strategies for both OM and CSM prediction (Supplementary Figure S3). These findings suggest that the RSF models may provide clinically useful risk stratification beyond discrimination performance alone.

Formal pairwise comparisons of Harrell’s C-index between survival models were additionally performed using paired nonparametric bootstrap resampling of the held-out test set (Supplementary Table S7). RSF showed significantly higher C-index values than the Cox model for both OM and CSM prediction. However, the differences in C-index between RSF and XGBoost-based survival models were not statistically significant, suggesting broadly comparable discrimination performance among the best-performing ML models.

For 2-year survival prediction, the RSF model achieved the best overall performance, with an accuracy of 0.750, AUC of 0.820, recall of 0.787, mean precision of 0.744, and F1-score of 0.764 (Table 4). This was followed by XGBoost and LightGBM. For 3-year survival prediction, RSF again demonstrated the highest accuracy (0.751), AUC (0.822), and mean precision (0.690). However, XGBoost achieved the best recall (0.493) and F1-score (0.545), metrics that are particularly important for evaluating performance in imbalanced classification tasks. Given the balanced nature of the F1-score in reflecting both precision and recall, XGBoost was considered more generalizable for long-term survival prediction.

In both 2-year and 3-year predictions, all ML models outperformed traditional methods, such as logistic regression and Cox proportional hazards modeling, highlighting the potential of ML approaches for more accurate survival classification in patients with CRPC.

### 3.3. Attribute Weight

**Figure 2:**
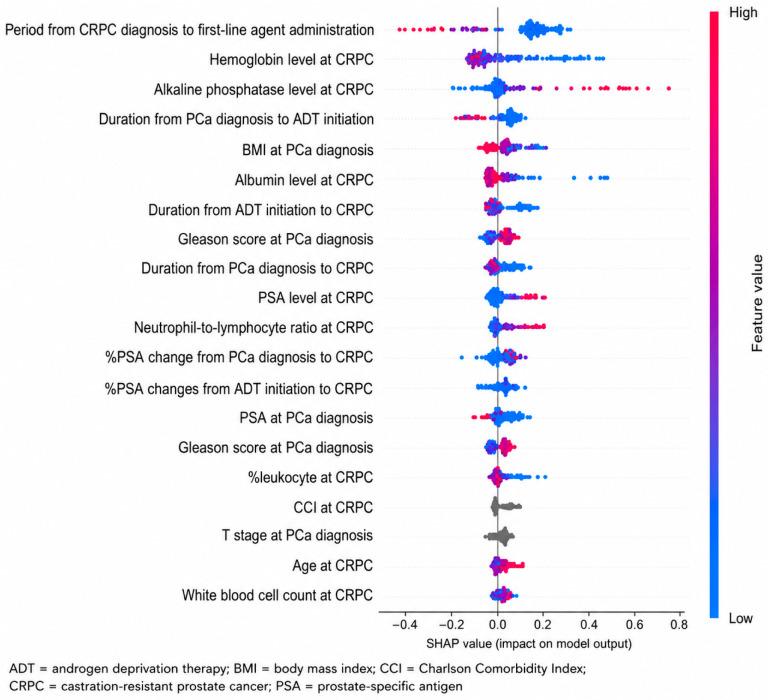
SHAP summary plot for XGB regression model based on CSM.

**Figure 3:**
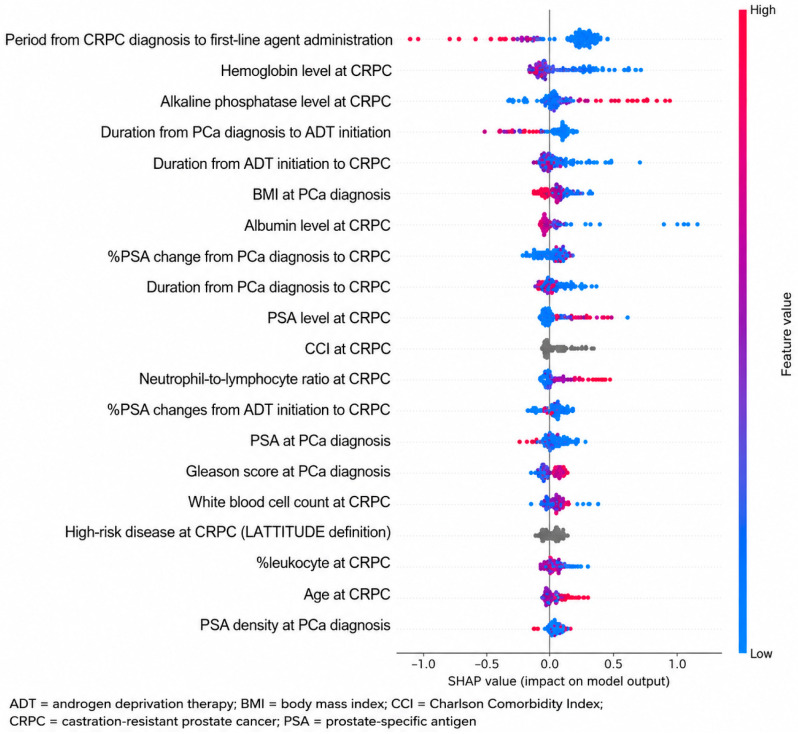
SHAP summary plot for XGB regression model based on OM.

The SHAP summary plots (Figure 2 and Figure 3) illustrate the overall impact and distribution of individual risk variables, ranked in descending order of importance. In the CSM model (Figure 2), the most influential feature was the time interval from CRPC diagnosis to initiation of first-line therapy. Other top-ranked predictors included baseline hemoglobin and alkaline phosphatase (ALP) levels at the time of CRPC diagnosis, as well as the duration from initial PCa diagnosis to ADT initiation. Notably, these same variables were also identified as the most influential features in the corresponding OM model (Figure 3), indicating their consistent prognostic relevance across different survival endpoints.

## 4. Discussion

The quality of data preparation plays a critical role in the performance of ML algorithms, especially in CRPC survival prediction, where outcomes are influenced by a complex interplay of clinical and biological factors. A key strength of our study is the use of one of the largest and most comprehensive CRPC dataset to date, incorporating 46 clinical, laboratory, and pathological variables spanning the full disease landscape, from initial PCa diagnosis to death. Our study expands the growing literature on ML-based prognostic modeling in CRPC by comparatively evaluating multiple interpretable ML approaches in a heterogeneous real-world cohort [22,23].

Although numerous studies have explored survival prediction in PCa, most have focused on localized disease, often utilizing deep learning models trained on large and relatively homogeneous patient populations [24]. In contrast, our study uniquely targeted a CRPC population, which is typically characterized by small sample sizes and greater clinical heterogeneity. For instance, Dai et al. reported a C-index of up to 0.85 using deep learning models for localized PCa cohorts, benefiting from more uniform disease characteristics and larger data volumes [25]. Despite working with a more complex dataset, our ML models achieved robust predictive performance, with C-index values of up to 0.77. These results demonstrate the potential of ML to achieve robust predictive performance in a substantially more complex clinical setting. Our findings further suggest that future applications of deep learning, tailored to the intricacies of advanced disease, may further improve prognostic accuracy.

By incorporating a broad range of input variables and comparing multiple ML algorithms, we demonstrated that survival prediction for CRPC can be significantly improved compared with traditional statistical methods. Our top-performing models achieved C-indices of 0.772 for CSM and 0.771 for OM in the test set, substantially higher than the C-index of 0.67 reported by Moreira et al., who used Cox proportional hazards models to predict OM in a smaller dataset comprising 205 patients and 14 variables [26]. These findings highlight the importance of both dataset comprehensiveness and algorithmic methodology in achieving superior predictive performance.

Saito et al. reported ML-based survival prediction models for PCa patients treated with ADT, achieving a C-index of 0.74 using RSF. Although their survival tree model achieved a higher C-index of 0.85 in metastatic PCa patients, it lacked generalizability to non-metastatic cases [27]. In contrast, our study included a broader CRPC population and evaluated multiple ML algorithms, including RSF, XGBoost, LightGBM, and logistic regression, allowing for a more comprehensive comparison. Although the C-index values of our models were relatively lower than those previously reported, our models demonstrated superior overall performance across both metastatic and non-metastatic CRPC populations, supporting broader applicability in real-world clinical practice.

In our analysis, XGBoost demonstrated higher performance in the validation set; however, it showed a relative decline in the test set, indicating a tendency toward overfitting. This reflects a limitation of XGBoost, which may capture noise or idiosyncrasies of the training set rather than generalizable prognostic patterns. Overfitting not only diminishes predictive stability but may also limit clinical utility when models are applied to external populations. To mitigate this issue, we applied several strategies, including hyperparameter optimization using grid search within a 10-fold cross-validation framework, application of regularization parameters (e.g., L1 and L2) embedded within algorithms such as XGBoost and LightGBM, and evaluation of model generalizability through performance assessment in an independent test set. Additional approaches, such as stricter regularization, early stopping to prevent over-training, or dimensionality reduction by prioritizing features with stronger prognostic value, could be considered in future studies to further reduce the risk of overfitting. To balance accuracy with generalizability, RSF and LightGBM were evaluated alongside XGBoost as part of a complementary modeling approach. Ultimately, the most critical step will be external validation using independent cohorts, which would confirm model robustness and support broader generalizability.

A key advantage of our approach is the integration of SHAP, which improved the interpretability of our ML models by quantifying the individual contribution of each input variable. Among the top-ranked predictors for both CSM and OM were the time interval from CRPC diagnosis to initiation of first-line systemic therapy, as well as baseline hemoglobin and ALP levels at the time of CRPC diagnosis. Notably, traditional prognostic indicators such as age, Gleason grade, and baseline PSA contributed minimally to the models, suggesting a shift in prognostic relevance toward more dynamic treatment-related and biochemical variables in the advanced disease setting. However, the interval from CRPC diagnosis to initiation of first-line therapy should be interpreted with caution. This variable may be affected by confounding by indication and potential reverse causality because patients with rapidly progressive, symptomatic, or high-burden disease are more likely to receive immediate systemic treatment, whereas patients with a more indolent disease course may undergo observation before treatment initiation. Therefore, this feature should not be regarded as a purely baseline prognostic factor or a directly actionable treatment-delay variable, but rather as a composite marker reflecting disease aggressiveness, clinical decision-making, and real-world treatment patterns. In addition, SHAP-derived feature importance should be interpreted as an association within the predictive model rather than evidence of a causal relationship.

Prior studies have identified anemia and elevated or rapidly increasing ALP levels as strong independent predictors of poor prognosis in mCRPC, particularly in large cohorts treated with docetaxel or cabazitaxel [28,29]. Imaging-based investigations have also emphasized the prognostic significance of ALP kinetics [30]. In contrast, conventional variables such as Gleason grade and PSA appeared to lose prognostic relevance after progression to the castration-resistant state. This observation aligns with recent meta-analyses and PSMA–PET–based studies, which similarly reported limited or no survival impact of Gleason grade in the mCRPC setting [31,32]. Taken together, these data support a paradigm shift in which survival prediction models in advanced PCa may benefit from prioritizing systemic and bone turnover markers, such as Hb and ALP, over traditional histopathologic or laboratory prognosticators.

Several limitations should be noted. First, the lack of an external validation cohort restricts the generalizability of our findings. However, the inclusion of patients managed by eight practicing uro-oncologists across two academic tertiary referral hospitals, with treatment decisions and clinical practice patterns determined independently by each physician, may partially enhance the heterogeneity and real-world representativeness of the dataset. Second, our dataset spans an 18-year period (2005–2022) during which substantial advancements in systemic therapies for CRPC occurred. During this period, novel agents such as androgen receptor-axis targeted therapies (e.g., abiraterone, enzalutamide), cabazitaxel, and various combination or sequential strategies were gradually introduced. These therapeutic shifts may have significantly influenced survival outcomes and consequently affected the predictive performance of our models. Era-based sensitivity analyses to account for temporal heterogeneity were considered; however, irregular timing of therapeutic changes and resulting heterogeneity within subgroups limited the feasibility and statistical reliability of such analyses. Third, recently approved treatments not captured in our cohort, including darolutamide, pembrolizumab, and rucaparib, may further affect contemporary prognostic patterns. Future studies incorporating these agents and performing external validation in contemporary cohorts will be important to strengthen predictive accuracy, clinical relevance, and transportability. Finally, although calibration analyses (calibration plots, Brier score, and integrated Brier score) and clinical utility assessment using decision curve analysis were incorporated, further prospective validation in independent external cohorts remains necessary before real-world clinical implementation.

## 5. Conclusions

Using a large and comprehensive dataset along with multiple ML algorithms, we demonstrated that XGBoost and RSF can substantially outperform traditional statistical methods in predicting CSM and OM in patients with CRPC. Importantly, the application of SHAP improved model interpretability by identifying clinically meaningful prognostic factors, which may support individualized treatment planning. Future studies should focus on model refinement, incorporation of emerging therapeutic agents, and external validation to ensure broad clinical applicability and successful translation into real-world practice.

## Figures and Tables

**Table 1:** Clinical, laboratory, and pathological characteristics.

Number	801
At initial PCa diagnosis	
Body mass index (kg/m^2^)	24.0 (21.6–25.7)
PSA (ng/mL)	65.6 (18.2–280.9)
PSA density (ng/mL/cc)	1.58 (0.47–6.21)
Gleason score	
≤7	131 (16.4%)
≥8	670 (83.6%)
Extent of metastasis	
Bone	439 (54.7%)
Lymph node	283 (35.3%)
Lung	43 (5.4%)
Liver	13 (1.6%)
NCCN risk category	
Intermediate	36 (4.5%)
High	765 (95.5%)
Clinical T stage	
≤T2	115 (14.4%)
≥T3	686 (85.6%)
Clinical N1 stage	
N0	395 (49.3%)
N1	406 (50.7%)
Clinical M1 stage	
M0	356 (44.4%)
M1	445 (55.6%)
Type of definitive treatment	
Radical prostatectomy	96 (12.0%)
Radiation therapy with or without ADT	243 (30.3%)
ADT alone	462 (57.7%)
PSA level at ADT initiation	46.6 (10.0–255.5)
Duration from ADT administration to CRPC (months)	0.0 (0.0–3.0)
At CRPC progression	
Age (years)	70.0 (65.0–76.0)
Presence of SPM	68 (8.5%)
Presence of SPM before CRPC progression	50 (6.2%)
Comorbidity	
Hypertension	332 (41.4%)
Diabetes mellitus	162 (20.2%)
Pulmonary tuberculosis history	29 (3.6%)
Liver cirrhosis	5 (0.6%)
Cerebrovascular disease	27 (3.4%)
CCI	
≤1	623 (77.8%)
≥2	178 (22.2%)
ECOG performance score	
≤1	738 (92.1%)
≥2	63 (7.9%)
Period from CRPC diagnosis to first treatment (months)	0.0 (0.0–4.0)
Period from PCa diagnosis to CRPC diagnosis (months)	28.0 (12.0–56.0)
Period from ADT initiation to CRPC diagnosis (months)	22.0 (10.0–47.0)
Metastatic site	
Bone	615 (76.7%)
Lymph node	295 (36.8%)
Lung	71 (8.9%)
Liver	40 (5.0%)
Number of metastatic sites	
<3 lesions	131 (16.3%)
≥3 lesions	484 (60.3%)
High-risk disease (LATTITUDE definition)	445 (55.6%)
High-volume disease (CHAARTED definition)	517 (64.5%)
PSA level at CRPC diagnosis	17.5 (4.7–76.6)
%PSA change at CRPC diagnosis	
From PCa diagnosis (%)	−72.8 (−94.2–14.6)
From ADT initiation (%)	−60.5 (−171.6–−0.93)
Laboratory data	
Hemoglobin (g/dL)	12.5 (11.4–13.3)
WBC count (/μL)	5985.0 (4937.0–7272.0)
Lymphocyte (/μL)	1610.0 (140.0–2110.0)
Neutrophil (/μL)	3620.0 (2800.0–4700.0)
Neutrophil-to-lymphocyte ratio	
<2	436 (54.4%)
≥2	365 (45.6%)
Cholesterol (mmol/L)	176.0 (148.0–204.0)
Albumin (g/dL)	4.2 (3.9–4.5)
Alkaline phosphatase (IU/L)	94.0 (69.0–163.8)
Follow-up duration, median	24.0 (12.0–43.0)
Cancer-specific death	566 (70.6%)
Overall death	588 (73.4%)

Data are number (%) and median (interquartile range). ADT = androgen-deprivation therapy; CCI = Charlson Comorbidity Index; CRPC = castration-resistant prostate cancer; ECOG = Eastern Cooperative Oncology Group; NCCN = National Comprehensive Cancer Network; PCa = prostate cancer; PSA = prostate-specific antigen; SPM = second primary malignancy; WBC = white blood cell.

**Table 2:** Summary of 2-year and 3-year survivals in patients with CRPC.

	Cancer-Specific Survival (%)	Overall Survival (%)
2-year	56.5%	54.3%
3-year	37.2%	34.3%

**Table 3:** Performance of regression models.

		Cox	RSF	XGB	XGB (with Its Own Imputation)
Valid score	CSM	0.685	0.764	0.761	0.771
	95% CI	0.656–0.714	0.698–0.830	0.695–0.827	0.706–0.836
	OM	0.6934	0.771	0.770	0.773
	95% CI	0.665–0.722	0.706–0.836	0.705–0.835	0.708–0.838
Test score	CSM	0.6210	0.772	0.770	0.753
	95% CI	0.590–0.652	0.707–0.837	0.705–0.835	0.686–0.820
	OM	0.6130	0.771	0.756	0.765
	95% CI	0.584–0.642	0.706–0.836	0.689–0.823	0.699–0.831

CI = confidence interval; CSM = cancer-specific mortality; OM = overall mortality; RSF = random survival forest; XGB = extreme gradient boosting.

**Table 4:** Performance of classification models.

	Model	Accuracy	AUC	Recall	Precision	F1-Score
2-year survival	Logistic Regression	0.6356	0.7271	0.6818	0.6353	0.6528
	LightGBM	0.7107	0.8074	0.7442	0.7078	0.7236
	XGB	0.7198	0.8138	0.7586	0.7151	0.7350
	RandomForest	0.7504	0.8196	0.7868	0.7443	0.7640
3-year survival	Logistic Regression	0.7183	0.7069	0.3105	0.5958	0.3993
	LightGBM	0.7432	0.8017	0.4817	0.6275	0.5375
	XGB	0.7485	0.7861	0.4925	0.6246	0.5452
	RandomForest	0.7506	0.8224	0.3905	0.6903	0.4818

LightGBM = light gradient-boosting machine; XGB = extreme gradient boosting.

## Data Availability

The data are not publicly available due to privacy and ethical restrictions. Access to the de-identified data is restricted and may be available only under specific conditions with Institutional Review Board approval.

## Supplementary Materials

The following supporting information can be downloaded at: https://www.mdpi.com/article/10.3390/cancers18121866/s1, Figure S1: Machine learning preprocessing and model development workflow; Figure S2: Calibration plots of the RSF models for OM and CSM on the held-out test set at 24- and 36-month time points; Figure S3: Decision curve analysis of the RSF models for OM and CSM prediction on the held-out test set at 24- and 36-month time points; Table S1: Hyperparameter settings and ranges for regression models.; Table S2: Hyperparameter settings and ranges for classification models; Table S3: Distribution of systemic agents administered according to treatment lines; Table S4: Summary of missingness and imputation procedures for variables included in the machine learning workflow; Table S5: Implementation details and final hyperparameters of survival models; Table S6: Calibration and Clinical Utility Metrics of the Final RSF Models; Table S7: Pairwise bootstrap comparison of Harrell’s C-index between survival models; Table S8: Software Environment and Reproducibility Settings.

## Abbreviations

The following abbreviations are used in this manuscript:

ADT	Androgen deprivation therapy
ALP	Alkaline phosphatase
AUC	Area under the receiver operating characteristic curve
BMI	Body mass index
CCI	Charlson Comorbidity Index
CI	Confidence interval
CRPC	Castration-resistant prostate cancer
CSM	Cancer-specific mortality
CV	Cross-validation
ECOG	Eastern Cooperative Oncology Group
LightGBM	Light Gradient Boosting Machine
ML	Machine learning
NCCN	National Comprehensive Cancer Network
NLR	Neutrophil-to-lymphocyte ratio
OM	Overall mortality
OS	Overall survival
PCa	Prostate cancer
PCWG2	Prostate Cancer Working Group 2
PSA	Prostate-specific antigen
RSF	Random survival forest
SHAP	SHapley Additive exPlanations
XGB	Extreme gradient boosting
