# Supplementary material for: Multi-Model Machine Learning for Survival Predictions for Castration-Resistant Prostate Cancer
Source: Cancers (Basel). 2026 Jun 7;18(12):1866. doi: 10.3390/cancers18121866 (PMC13297037; doi:10.3390/cancers18121866)
Supplement: Supplementary file 1 [file cancers-18-01866-s001.zip › cancers-4312871-supplementary.pdf]

Supplementary Table S1. Hyperparameter settings and ranges for regression models.

	Hyperparameter	Range
RSF	n_estimator	10 – 500
	max_depth	1 – 40
	min_samples_split	2 – 30
	min_samples_leaf	1 – 40
	max_features	sqrt, log2, None
XGB	learning_rate	0.01 – 0.3
	max_depth	3 – 40
	gamma	1e–9 – 1.0
	subsample	0.5 – 1.0
	colsample_bytree	0.5 – 1.0
	lambda	1e–3, 10
	alpha	1e–3, 1

RSF = random survival forest; XGB = extreme gradient boosting

Supplementary Table S2. Hyperparameter settings and ranges for classification models.

	Hyperparameter	Range
LightGBM	n_estimators	100, 200, 300, 400, 500
	max_depth	-1, 3, 6, 9
	learning_rate	0.01, 0.1, 0.2, 0.3
	num_leaves	20, 31, 40, 50
	min_child_samples	10, 20, 30, 40
	subsample	0.5, 0.7, 0.9, 1.0
	colsample_bytree	0.5, 0.7, 0.9, 1.0
	reg_alpha	0, 0.1, 0.5, 1.0
	reg_lambda	0, 0.1, 0.5, 1.0
Random forest	n_estimators	100, 200, 300, 400, 500
	max_depth	3, 6, 9, 12, None
	min_samples_split	2, 5, 10
	min_samples_leaf	1, 2, 4
	bootstrap	True, False
	max_features	auto, sqrt, log2
XGB	n_estimators	100, 200, 300, 400, 500

max_depth	3, 6, 9, 12
learning_rate	0.01, 0.1, 0.2, 0.3
subsample	0.5, 0.7, 0.9, 1.0
colsample_bytree	0.5, 0.7, 0.9, 1.0
gamma	0, 0.1, 0.2, 0.3
min_child_weight	1, 3, 5

LightGBM = light gradient-boosting machine; XGB = extreme gradient boosting

Supplementary Table S3. Distribution of systemic agents administered according to treatment lines.

Agents	1st-line	2nd-line	3rd-line	4th-line
Cabazitaxel, n (%)	1 (0.2%)	11 (2.4%)	89 (43.2%)	14 (26.4%)
Docetaxel, n (%)	541 (67.5%)	122 (26.1%)	9 (4.4%)	2 (3.8%)
Enzalutamide, n (%)	161 (20.1%)	238 (51.0%)	52 (25.2%)	12 (22.6%)
Abiraterone, n (%)	98 (12.2%)	96 (20.6%)	55 (26.7%)	20 (37.7%)
Olaparib, n (%)	0 (0.0%)	0 (0.0%)	1 (0.5%)	5 (9.4%)
Total, n	801	467	206	53

Supplementary Table S4. Summary of missingness and imputation procedures for variables included in the machine learning workflow.

Variable	Variable type	N missing	Missing (%)	Imputation method	Model inclusion
Number	Identifier	0	0	Not imputed	No
Body mass index at baseline	Continuous	56	7	Iterative (BayesianRidge)	Yes
PSA level at baseline	Continuous	8	1	Iterative (BayesianRidge)	Yes
Gleason score at baseline	Categorical	12	1.5	Most frequent	Yes
Gleason score, primary at baseline	Categorical	12	1.5	Most frequent	Yes
Gleason score, secondary at baseline	Categorical	12	1.5	Most frequent	Yes
Gleason total, at baseline	Categorical	12	1.5	Most frequent	Yes
Prostate volume	Continuous	108	13.5	Iterative (BayesianRidge)	Yes
PSA density	Continuous	108	13.5	Iterative (BayesianRidge)	Yes
T stage at baseline	Categorical	13	1.6	Most frequent	Yes
N stage at baseline	Categorical	2	0.2	Most frequent	Yes
M stage at baseline	Categorical	0	0	No missing values	Yes
Bone metastasis at baseline	Categorical	0	0	No missing values	Yes
Lung metastasis at baseline	Categorical	0	0	No missing values	Yes
Liver metastasis at baseline	Categorical	0	0	No missing values	Yes
Lymph node metastasis at baseline	Categorical	0	0	No missing values	Yes
Risk category at baseline	Categorical	4	0.5	Most frequent	Yes
Curative treatment modality	Categorical	0	0	No missing values	Yes
ADT initiation, date	Date	801	100	Not imputed	No
Initial ADT duration	Continuous	1	0.1	Iterative (BayesianRidge)	Yes
PSA at ADT initiation	Continuous	24	3	Iterative (BayesianRidge)	Yes
%PSA change after ADT initiation	Continuous	28	3.5	Iterative (BayesianRidge)	Yes
CRPC onset duration	Continuous	0	0	No missing values	Yes
ADT initiation to CRPC duration	Continuous	2	0.2	Iterative (BayesianRidge)	Yes

Bone metastasis at CRPC	Categorical	0	0	No missing values	Yes
Lung metastasis at CRPC	Categorical	0	0	No missing values	Yes
Liver metastasis at CRPC	Categorical	0	0	No missing values	Yes
Lymph node metastasis at CRPC	Categorical	0	0	No missing values	Yes
Bone metastasis volume (high vs. low)	Categorical	105	13.1	Most frequent	Yes
Risk (LATTITUDE criteria)	Categorical	0	0	No missing values	Yes
Risk (CHAARTED criteria)	Categorical	0	0	No missing values	Yes
PSA at CRPC	Continuous	6	0.7	Iterative (BayesianRidge)	Yes
%PSA change after CRPC	Continuous	14	1.7	Iterative (BayesianRidge)	Yes
%PSA change after ADT initiation	Continuous	29	3.6	Iterative (BayesianRidge)	Iterative (BayesianRidge)
Cholesterol level at CRPC	Continuous	98	12.2	Iterative (BayesianRidge)	Iterative (BayesianRidge)
Albumin level at CRPC	Continuous	64	8	Iterative (BayesianRidge)	Yes
ALP level at CRPC	Continuous	73	9.1	Iterative (BayesianRidge)	Yes
WBC level at CRPC	Continuous	71	8.9	Iterative (BayesianRidge)	Yes
Hemoglobin level at CRPC	Continuous	71	8.9	Iterative (BayesianRidge)	Yes
Neutrophil count at CRPC	Continuous	98	12.2	Iterative (BayesianRidge)	Yes
Lymphocyte count at CRPC	Continuous	98	12.2	Iterative (BayesianRidge)	Yes
NL ratio at CRPC	Continuous	98	12.2	Iterative (BayesianRidge)	Yes
Second primary malignancy (baseline)	Categorical	3	0.4	Most frequent	Yes
Second primary malignancy (at CRPC)	Categorical	3	0.4	Not imputed	No
Age at CRPC	Continuous	7	0.9	Iterative (BayesianRidge)	Yes
Hypertension at CRPC	Categorical	9	1.1	Most frequent	Yes
Diabetes mellitus at CRPC	Categorical	8	1	Most frequent	Yes
Tuberculosis at CRPC	Categorical	8	1	Most frequent	Yes
Liver cirrhosis at CRPC	Categorical	8	1	Most frequent	Yes
Cerebrovascular disease at CRPC	Categorical	8	1	Most frequent	Yes

Charlson comorbidity index at CRPC	Continuous	4	0.5	Iterative (BayesianRidge)	Yes
ECOG performance score at CRPC	Categorical	4	0.5	Most frequent	Yes
CRPC, date	Date	801	100	Not imputed	No
CRPC diagnosis to 1st treatment duration	Continuous	21	2.6	Not imputed	No
1st treatment	Categorical	0	0	Not imputed	No
1st treatment, date	Date	0	0	Not imputed	No
PSA at 1st treatment	Continuous	3	0.4	Iterative (BayesianRidge)	Yes
Duration of 1st treatment	Continuous	0	0	Not imputed	No
2nd treatment	Categorical	334	41.7	Not imputed	No
2nd treatment, date	Date	334	41.7	Not imputed	No
PSA at 2nd treatment	Continuous	336	41.9	Not imputed	No
Duration of 2nd treatment	Continuous	336	41.9	Not imputed	No
3rd treatment	Categorical	596	74.4	Not imputed	No
3rd treatment, date	Date	596	74.4	Not imputed	No
PSA at 3rd treatment	Continuous	597	74.5	Not imputed	No
Duration of 3rd treatment	Continuous	602	75.2	Not imputed	No
4th treatment	Categorical	753	94	Not imputed	No
4th treatment, date	Date	754	94.1	Not imputed	No
PSA at 4th treatment	Continuous	753	94	Not imputed	No
Duration of 4th treatment	Continuous	754	94.1	Not imputed	No
5th treatment	Categorical	799	99.8	Not imputed	No
5th treatment, date	Date	800	99.9	Not imputed	No
PSA at 5th treatment	Continuous	799	99.8	Not imputed	No
Duration of 5th treatment	Continuous	799	99.8	Not imputed	No
Last follow-up, date	Date	801	100	Not imputed	No
CRPC to last follow-up duration	Survival time	0	0	Not imputed	No

Overall mortality	Outcome	3	0.4	Not imputed	No
Cancer-specific mortality	Outcome	3	0.4	Not imputed	No

Supplementary Table S5. Implementation details and final hyperparameters of survival models.

Part A. XGBoost survival implementation

Item	Description
Implementation	xgboost.train API
Objective	survival:cox
Censoring handling	Positive time for events, negative time for censored observations
Eval metric	cox-nloglik
Tree method	hist
Max boosting rounds	1000
Early stopping	50 rounds
Hyperparameter tuning	Optuna TPE sampler
CV strategy	10-fold CV
Random seed	42

Part B. Final hyperparameters (XGB)

Endpoint	Model-matrix features	Best iteration	max_depth	eta	subsample	ambda	alpha	Test C-index
OM	97	97	5	0.050640	0.830500	9.937043	0.000479	0.756
CSM	97	206	5	0.019731	0.640868	0.619993	0.000399	0.791

Part C. Final hyperparameters (XGB with its own imputation)

Endpoint	Model-matrix features	Best iteration	max_depth	eta	subsample	lambda	alpha	Test C-index
OM	50	90	5	0.036651	0.822288	2.971037	0.001874	0.760
CSM	50	106	5	0.035282	0.602747	0.713021	0.001807	0.775

Part D. Cox PH assumption

Endpoint	PH assumption result
OM	3/97 terms violated PH assumption
CSM	1/97 terms violated PH assumption

Abbreviations: CSM = cancer-specific mortality; OM = overall mortality

Supplementary Table S6. Calibration and Clinical Utility Metrics of the Final RSF Models.

Endpoint	Horizon	C-index	Brier score	Integrated Brier score
OM	24 months	0.762	0.165	0.162
OM	36 months	0.762	0.155	0.162
CSM	24 months	0.791	0.171	0.166
CSM	36 months	0.791	0.159	0.166

Abbreviations: CSM = cancer-specific mortality; OM = overall mortality; RSF = Randon Survival Forests

Supplementary Table S7. Pairwise bootstrap comparison of Harrell's C-index between survival models

**Endpoint**	**Comparison**	**Δ C-index**	**95% CI**	***P*-value**	**Holm-adjusted *p*-value**
OM	RSF vs Cox	0.066	0.019 to 0.117	*0.0060*	*0.0360*
OM	RSF vs XGB	0.006	-0.018 to 0.029	*0.5634*	*1.0000*
OM	RSF vs XGB (with its own imputation)	0.002	-0.023 to 0.025	*0.8651*	*1.0000*
OM	XGB vs Cox	0.060	0.013 to 0.108	*0.0100*	*0.0400*
OM	XGB (with its own imputation) vs Cox	0.063	0.020 to 0.113	*0.0080*	*0.0400*
OM	XGB vs XGB (with its own imputation)	-0.004	-0.019 to 0.010	*0.6094*	*1.0000*
CSM	RSF vs Cox	0.066	0.019 to 0.117	*0.0060*	*0.0360*
CSM	RSF vs XGB	0.006	-0.018 to 0.029	*0.5634*	*1.0000*
CSM	RSF vs XGB (with its own imputation)	0.002	-0.023 to 0.025	*0.8651*	*1.0000*
CSM	XGB vs Cox	0.060	0.013 to 0.108	*0.0100*	*0.0400*
CSM	XGB (with its own imputation) vs Cox	0.063	0.020 to 0.113	*0.0080*	*0.0400*
CSM	XGB vs XGB (with its own imputation)	-0.004	-0.019 to 0.010	*0.6094*	*1.0000*

Δ C-index was calculated as the C-index of the first model minus that of the second model. Pairwise comparisons were performed using paired nonparametric bootstrap resampling of the held-out test set. Holm correction was applied for multiple pairwise comparisons within each endpoint.

Abbreviations: CSM = cancer-specific mortality; OM = overall mortality; RSF = Randon Survival Forests; XGB = XGBoost

Supplementary Table S8. Software Environment and Reproducibility Settings

Category	Description
Programming language	Python 3.x
Operating environment	Windows/Linux (whatever used)
Random seed	42
Hyperparameter optimization	Optuna (TPE sampler)
Cross-validation	10-fold cross-validation
Main libraries	scikit-learn, xgboost, lifelines, scikit-survival, shap
Imputation	IterativeImputer (BayesianRidge), SimpleImputer
Survival metric	Harrell's C-index
Classification metrics	AUC, accuracy, recall, precision, F1-score
Early stopping	early_stopping_rounds = 50
SHAP implementation	TreeExplainer

Abbreviations: AUC = Area Under the Curve, SHAP = SHapley Additive exPlanations; TPE = Tree-structured Parzen Estimator

Supplementary Figure S1. Machine learning preprocessing and model development workflow

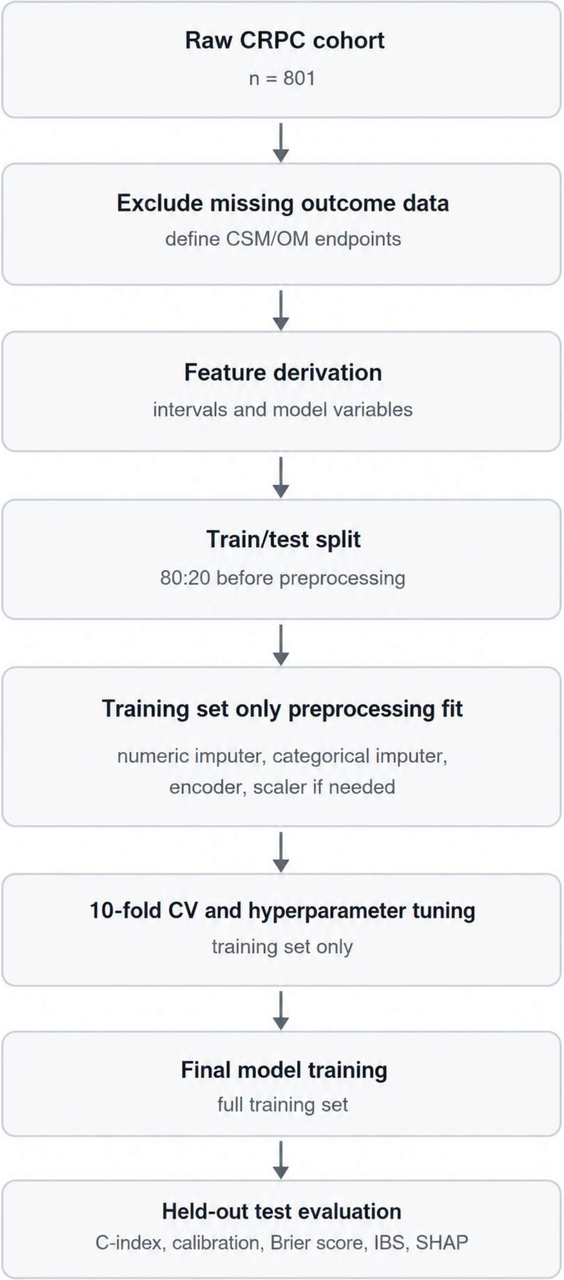

Supplementary Figure S2. Calibration plots of the RSF models for OM and CSM on the held-out test set at 24- and 36-month time points.

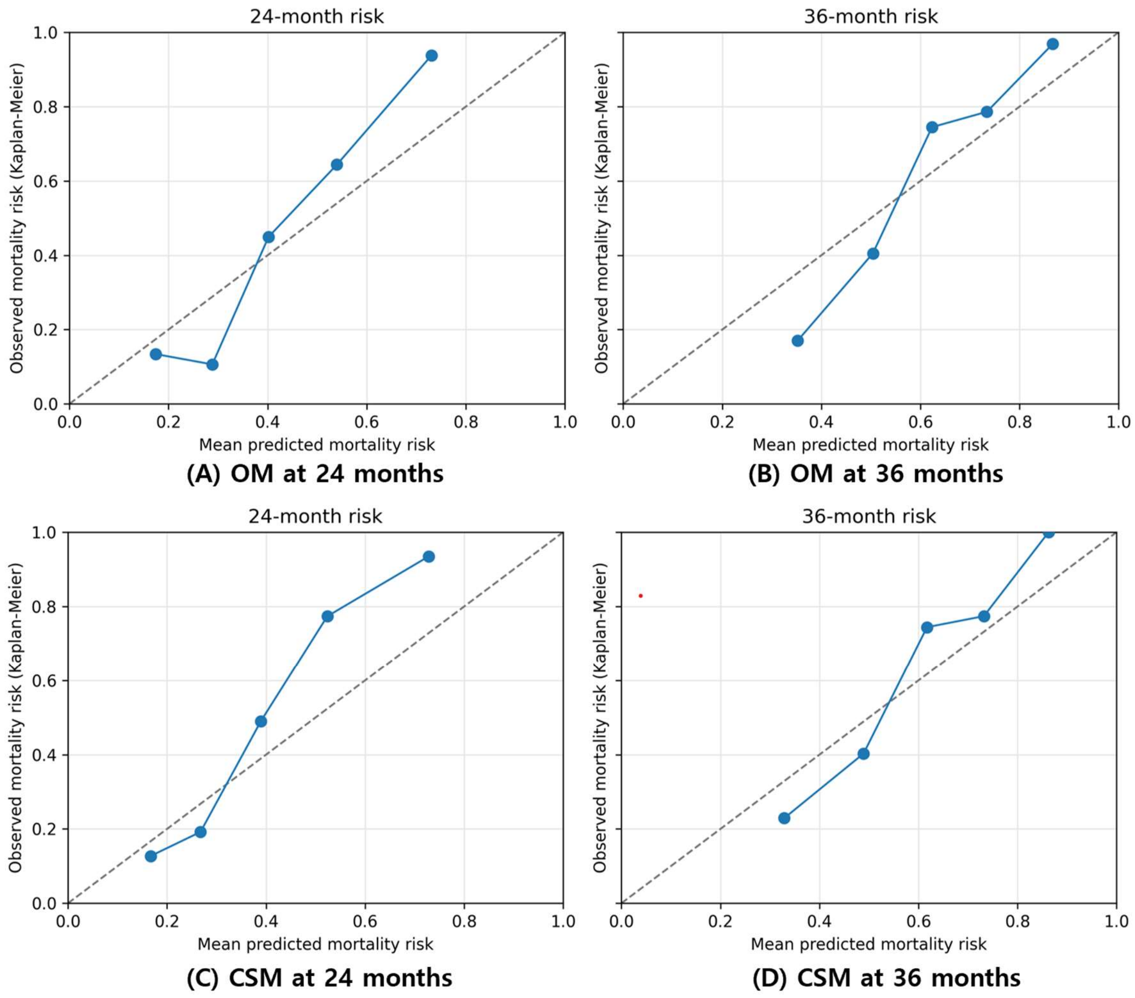

Supplementary Figure S2A. Calibration plot for 24-month overall mortality prediction.
Supplementary Figure S2B. Calibration plot for 36-month overall mortality prediction.
Supplementary Figure S2C. Calibration plot for 24-month cancer-specific mortality prediction.
Supplementary Figure S2D. Calibration plot for 36-month cancer-specific mortality prediction.

Abbreviations: CSM = cancer-specific mortality; OM = overall mortality; RSF = Randon Survival Forests

Supplementary Figure S3. Decision curve analysis of the RSF models for OM and CSM prediction on the held-out test set at 24- and 36-month time points.

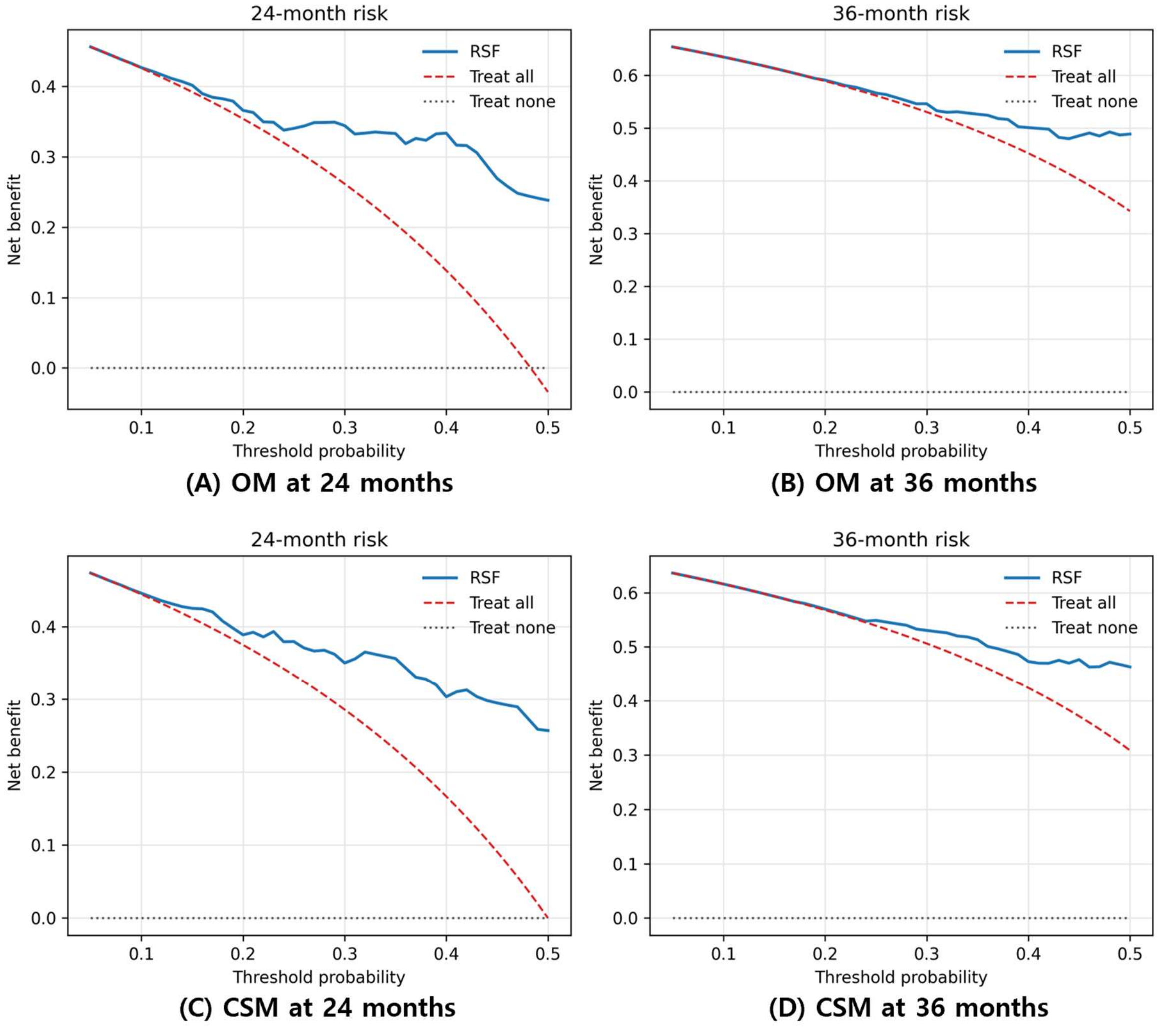

Supplementary Figure S3A. Decision curve analysis for 24-month overall mortality prediction.
Supplementary Figure S3B. Decision curve analysis for 36-month overall mortality prediction.
Supplementary Figure S3C. Decision curve analysis for 24-month cancer-specific mortality prediction.
Supplementary Figure S3D. Decision curve analysis for 36-month cancer-specific mortality prediction.

Abbreviations: CSM = cancer-specific mortality; OM = overall mortality; RSF = Randon Survival Forests
